# Correlation between clinical and laboratory parameters and early pregnancy loss in assisted reproductive technology cycles: A cross-sectional study

**DOI:** 10.18502/ijrm.v20i8.11757

**Published:** 2022-09-06

**Authors:** Fatemeh Emami, Maryam Eftekhar, Samaneh Jalaliani

**Affiliations:** ^1^Department of Obstetrics and Gynecology, Research and Clinical Center for Infertility, Shahid Sadoughi University of Medical Sciences, Yazd, Iran.; ^2^Abortion Research Center, Yazd Reproduction Sciences Institute, Shahid Sadoughi University of Medical Sciences, Yazd, Iran.

**Keywords:** Abortion, Pregnancy, Embryo transfer, Assisted reproductive techniques, Anti-Müllerian hormone.

## Abstract

**Background:**

The miscarriage rate after pregnancy resulting from assisted reproductive technology (ART) is about 20%, roughly half of which is biochemical. The correlations between the number and quality of oocytes, estradiol level and early pregnancy loss have not been fully clarified.

**Objective:**

This study aimed to examine the clinical and laboratory parameter effects on early abortion in ART cycles.

**Materials and Methods:**

In this cross-sectional study, 408 women who were ART candidates and were referred to the Yazd Infertility and Research Center, Yazd, Iran during March 2017 to March 2020 participated. Women who had a fresh embryo transferred and who had a positive beta human chorionic gonadotropin serum test were included in the study. The Anti-Müllerian hormone (AMH) level, embryo quality, oocyte number, progesterone level, estradiol level, and maternal age were extracted from the medical records.

**Results:**

No significant difference was observed in the age, mean estradiol and progesterone levels on trigger day, number of MII oocytes, and embryo quality between the groups (p = 0.19, 0.42, 0.07, 0.34 and 0.20, respectively). No statistically significant difference was found between the 3 groups of AMH level (p = 0.20). After evaluation using logistic regression, the rate of negative clinical pregnancies was higher in the group with AMH < 1 ng/ml. However, this was not found to be statistically significant.

**Conclusion:**

We did not find any correlation between early abortion and AMH levels, embryo quality, oocyte number, progesterone level, estradiol level, or maternal age.

## 1. Introduction

Assisted reproductive techniques (ART) is an important treatment in many infertile couples to get pregnant. The ART pregnancy rate is about 40%, but the rate of delivery is still 20-30%, and 1 of the main reasons is miscarriage (1). The incidence of early pregnancy loss in patients receiving fresh embryos in ART is 14.9% (2) and is higher than the miscarriage incidence after natural conception (3). In natural conception many abortions occur before clinical pregnancy detection and a number of miscarriages that occur following spontaneous pregnancies may not be diagnosed at all. In contrast, after pregnancy following ART, most women have an accurate diagnosis and follow up of pregnancy (4). Therefore, these women who become pregnant through ART are the ideal population in which to study the risk factors for early abortion.

One of the main reasons for the higher incidence of early abortion among women who become pregnant through ART is that these women are often older prime parous women in comparison with women who get naturally pregnant (4, 5). Based on previous research, we know that the clinical pregnancy rate is significantly associated with the age of donors (3) and biological age is more crucial than chronological age for childbearing (6).

Ovarian reserve and quality of oocytes significantly decrease with aging. In addition, chromosomal abnormalities increase with ovarian reserve decline and advanced maternal age (7). Therefore, low levels of Anti-Müllerian hormone (AMH) may be associated with early abortion, poor pregnancy outcome, as well as increased pregnancy comorbidity such as preeclampsia (8-11). Moreover, serum AMH level may affect in vitro fertilization outcomes (12), but AMH cannot be used to predict the live birth rate (13).

Apart from maternal age and AMH, factors such as the levels of estradiol and progesterone on trigger day, number of retrieved oocytes, number or type of embryos transferred, and ovarian stimulation protocol may also be associated with spontaneous miscarriage (14-18).

The overall sample sizes of previous studies have been small, and the mentioned factors interact with each other, so there is still no consensus on the effects of these factors.

The purpose of this study was to evaluate various factors that may affect early spontaneous abortion after ART treatment.

## 2. Materials and Methods

### Study population

This cross-sectional study was conducted with 480 women who were ART candidates referred to Yazd Infertility and Research Center, Yazd, Iran during March 2017-March 2020. Women who were referred to the clinic, had fresh embryos transferred and became chemically pregnant, as shown by testing positive for beta human chorionic gonadotropin (HCG) serum, were enrolled in the study. Women who became pregnant with egg donation or uterine surrogacy, those with partners with severe male factor infertility and those with incomplete files were excluded. At the end, 408 women were examined.

Data were collected and analyzed on the participant's age at the time of ART, AMH during the year of ART, type and cause of infertility, characteristics of the treatment cycle including stimulation protocol (agonist or antagonist), type of trigger (HCG or gonadotropin releasing hormone agonist), the number of oocytes, type of fertilization (in vitro fertilization or intracytoplasmic sperm injection), the number of frozen embryos, the number of transferred embryos, and the pregnancy results (chemical and clinical pregnancy).

### Therapeutic protocol 

Ovarian stimulation was done according to the agonist, antagonist, or microdose protocol (19). The initial dose of gonadotropin with recombinant follicle stimulating hormone or human menopausal gonadotropin was determined based on age, body mass index, AMH, and antral follicle count ranging from 150-450 units/day. Once the size of the follicles reached more than or equal to 17 mm, the trigger was done with gonadotropin releasing hormone agonist or HCG. The puncture was performed after 36-40 hr and the embryo was transferred after 2-3 days.

Vaginal progestin started on the day of egg retrieval and continued for up to 14 days, and vaginal progestin continued for up to 8 wk if the beta HCG blood test was positive. Only the women with a positive pregnancy blood test were included in the study after 14 days following the embryo transfer. Luteal phase support was based on women's conditions which consisted of vaginal progestin in all women, and if they did not receive HCG on trigger day, they would have received HCG 5000 U on the embryo transfer day.

### Assessment of pregnancy

Chemical pregnancy was assessed with serum beta HCG 14 days after embryo transfer. Women were considered clinically pregnant if they had a positive blood beta HCG test. The fetal heart was examined using transvaginal sonography or transabdominal sonography, which was done 4 wk after a positive blood pregnancy test.

Early abortion was defined as when women had a positive chemical pregnancy but negative clinical pregnancy. Ectopic pregnancies were excluded from the study because the underlying cause was different from those of early abortion.

### Ethical considerations

The study protocol was approved by the Ethics Committee of the Research and Clinical Center for Infertility, Yazd, Iran (Code: IR.SSU.RSI.REC.1399.048). Also, written informed consent was obtained from all participants before the study.

### Statistical analysis

The SPSS software (Statistical Package for the Social Sciences, version 26, Chicago, IL, USA) was used to perform all the statistical analyses. The baseline characteristics were compared between the groups using the student's *t* test for continuous data and the Chi-square test for categorical data. A p-value < 0.05 was considered statistically significant. In addition, logistic regression was used for estimating the effect of each variable on pregnancy outcomes.

## 3. Results

In this study, 60 women out of 408 had negative clinical pregnancies and were categorized as having had an early abortion, and 348 women with positive clinical pregnancy were categorized in the positive clinical pregnancy group. The effects of the parameters such as maternal age, AMH, estradiol and progesterone levels, and the number of oocytes and embryo quality on early abortion and clinical pregnancy rates were assessed.

Table I shows the characteristics of the women studied, including age, duration of infertility, type of infertility (primary or secondary), cause of infertility, the protocol of ovulation induction, and type of ART (in vitro fertilization or intracytoplasmic sperm injection).

Table II shows the effects of the following studied parameters on the rate of clinical pregnancy: AMH in 3 different groups ( < 1 ng/ml; 1-2 ng/ml; > 2 ng/ml) (10), mean estradiol level, mean number of oocytes, mean progesterone level, and embryo quality in 3 different groups (A = high quality, B = intermediate quality, and C = low quality, where the criteria for embryo quality were based on the embryologist evaluation and cleavage-stage embryos scored as grade A, B, C, and D). Grade A embryos were those with equally sized homogenous blastomeres with no fragmentation. Grade B embryos had equally sized homogenous blastomeres with < 20% fragmentation. Grade C embryos had unequally sized blastomeres with 20-50% fragmentation. Grade D embryos were those with unequally sized blastomeres with > 50% fragmentation (20); these were not transferred. Considering AMH > 2 ng/ml as the reference group, the odds ratio of the AMH < 1 ng/ml group was estimated to be 1.59 (95% confidence interval = 0.87-2.91) based on logistic regression, which means the chance of early abortion was higher in the AMH < 1 ng/ml group.

**Table 1:** The characteristics of the participants


**Variables **		**Early abortion (n = 60)**	**Positive clinical pregnancy (n = 348)**	**P-value**
Age (yr)*		32.70 ± 4.69	31.81 ± 4.83	0.19
Duration of infertility (yr)*		7.00 ± 4.26	6.66 ± 4.53	0.60
Type of infertility**				
	**Primary**	43 (71.6)	273 (78.5)	
	**Secondary**	17 (28.4)	75 (21.5)	0.25
Cause of infertility**				
	**PCO**	4 (6.7)	34 (9.8)	
	**Male factor**	16 (26.7)	99 (28.5)	
	**Endometriosis**	4 (6.7)	12 (3.4)	
	**Unexplained**	11 (18.3)	54 (15.5)	
	**Mixed**	11 (18.3)	86 (24.7)	
	**Amenorrhea**	1 (1.7)	3 (0.9)	
	**Tubal factor**	2 (3.3)	7 (2.0)	
	**Ovarian factor**	11 (18.3)	53 (15.2)	0.77
Type of protocol**				
	**Antagonist**	45 (75.0)	216 (62.0)		
	**Agonist**	4 (6.7)	44 (12.6)		
	**Micro dose**	11 (18.3)	88 (25.4)		0.14
*Data presented as Mean ± Standard deviation, *t* test. **Data presented as n (%), Chi-square test. PCO: Polycystic ovary					

**Table 2:** Effects of AMH, embryo quality, MII oocytes, estradiol level, and progesterone level on clinical pregnancy


		**Early abortion (n = 60)**	**Positive clinical pregnancy (n = 348)**	**P-value**
AMH (ng/ml)*				
	**< 1**	7 (11.7)	57 (16.4)	
	**1-2**	21 (35.0)	85 (24.4)	
	** > 2**	32 (53.3)	206 (59.2)	0.20
Quality of embryo*				
	**A**	29 (48.3)	126 (36.2)		
	**B**	27 (45.0)	189 (54.3)	
	**C**	4 (6.7)	33 (9.5)	0.20
Estradiol (pg/ml)**		1317.77 ± 680.14	1418.38 ± 920.36		0.42
MII oocyte number**		5.56 ± 2.88	6.00 ± 3.31	0.34
Progesterone level (ng/ml)**		0.43 ± 0.56***	0.55 ± 0.45	0.07
*Data presented as n (%), Chi-square test. **Data presented as Mean ± Standard deviation, *t* test. ***Median = 0.45, Interquartile range = 0.2-0.65. AMH: Anti-Müllerian hormone, MII: Mature oocyte				

## 4. Discussion

This study was conducted with 408 women of whom 348 individuals had a positive clinical pregnancy, while 60 had a negative clinical pregnancy and thus an early abortion. The effects of age, estradiol level, progesterone level, the number of oocytes, AMH, and oocyte quality on clinical pregnancy were investigated.

The results showed no significant difference between the positive clinical pregnancy and early abortion groups in terms of the mean age range (p = 0.19). This is inconsistent with the results of 1 study which indicated that maternal age was a predictor of early abortion (5).

Moreover, the estradiol levels on the trigger day were compared between the positive clinical pregnancy group and the early abortion group. The mean estradiol level in the positive clinical pregnancy group was 1418.38 ± 920.36 pg/ml, and in the early abortion group was 1317.77 ± 680.14 pg/ml. No statistically significant difference was found between the 2 groups (p = 0.42). These results are similar to a study which showed that serum estradiol levels above the 90 ${}^{\text{th}}$ percentile on the trigger day using HCG were associated with a lower fertilization rate, but this estradiol level did not affect embryo growth, implantation, clinical pregnancy, or the early abortion rate (14).

The number of MII oocytes was another factor investigated for its importance in clinical pregnancy, but there was no significant difference between the 2 groups of positive clinical pregnancy and early abortion (p = 0.34). In a previous study it was found that an increased number of oocytes was associated with a reduced rate of clinical abortion, and patients with poor ovarian response and fewer oocytes were at a higher risk for clinical abortion (15), but early abortion was not related to the number of oocytes.

In the present study, embryo quality was assessed as another factor in early abortion. The women were divided into 3 groups (A, B and C), based on the embryo quality score. The rate of positive clinical pregnancies was 81.3%, 87.4% and 89.9%, respectively, and no statistically significant differences were found between the 3 groups (p = 0.20). In other words, embryo quality was not found to be an essential factor in early abortion. These study results are inconsistent with a previous study which showed that the quality of embryo was an influential factor in the early abortion rate (21).

Furthermore, the AMH level was evaluated as another factor potentially affecting the incidence of early abortion Patients were divided into 3 groups: group 1 with AMH < 1 ng/ml, group 2 with 1-2 ng/ml AMH, group 3 with AMH > 2 ng/ml, and the percentage of clinical pregnancies was assessed in each group. The results showed that the percentages of pregnancies in group 1, 2 and 3 were 89.1%, 80.2% and 86.6%, respectively. No statistically significant difference was found between the 3 groups (p = 0.20) so AMH was not a prognostic factor in early abortion and clinical pregnancy. After evaluation using logistic regression, the rate of negative clinical pregnancies was found to be higher in the group with AMH < 1 ng/ml. However, this was not statistically significant. Contrary to our results, 1 study has shown a relationship between low AMH and early abortion (8). But another study on the relationship between AMH level and early abortion found that lower AMH levels were not associated with a higher rate of early abortion (22).

The present study showed that the mean progesterone level on trigger day using HCG was 0.43 ± 0.56 ng/ml in the early abortion group and 0.55 ± 0.45 ng/ml in the positive clinical pregnancy group, but no statistically significant difference was found between the 2 groups (p = 0.07). Our results are similar to other studies which also showed that higher progesterone levels were not associated with spontaneous abortion (16, 18).

According to our study, prognostic factors that affect ART outcomes are not necessarily prognostic of the abortion rate. We found no correlation between early abortion and AMH levels, embryo quality, oocyte number, progesterone level, estradiol level, or maternal age.

## 5. Conclusion

We did not find any correlation between early abortion and AMH levels, embryo quality, oocyte number, progesterone level, estradiol level, or maternal age.

##  Conflict of Interest

The authors declare that there is no conflict of interest.
