# Corrigendum to “Correlation between clinical and laboratory parameters and early pregnancy loss in assisted reproductive technology cycles: A cross-sectional study” [Int J Reprod BioMed 2022; 20: 683-690]

**DOI:** 10.18502/ijrm.v20i10.12272

**Published:** 2022-11-02

**Authors:** Fatemeh Emami, Maryam Eftekhar, Samaneh Jalaliani

The authors have been informed of an error that occurred on page 684 in which the number of participants should be replaced with 408 instead of 480. On behalf of the author, the publisher wishes to apologize for this error. The online version of article has been updated on 23 October 2022 and can be found at https://doi.org/10.18502/ijrm.v20i8.11757.

